# Uncovering recent progress in nanostructured light-emitters for information and communication technologies

**DOI:** 10.1038/s41377-021-00598-3

**Published:** 2021-07-29

**Authors:** Frédéric Grillot, Jianan Duan, Bozhang Dong, Heming Huang

**Affiliations:** 1 LTCI, Institut Polytechnique de Paris, Télécom Paris, 19 place Marguerite Perey, 91120 Palaiseau, France; 2 Center for High Technology Materials, University of New-Mexico, 1313 Goddard St SE, Albuquerque, NM 87106 USA; 3 State Key Laboratory on Tunable Laser Technology, School of Electronic and Information Engineering, Harbin Institute of Technology, Shenzhen, 518055 China

**Keywords:** Photonic devices, Semiconductor lasers

## Abstract

Semiconductor nanostructures with low dimensionality like quantum dots and quantum dashes are one of the best attractive and heuristic solutions for achieving high performance photonic devices. When one or more spatial dimensions of the nanocrystal approach the de Broglie wavelength, nanoscale size effects create a spatial quantization of carriers leading to a complete discretization of energy levels along with additional quantum phenomena like entangled-photon generation or squeezed states of light among others. This article reviews our recent findings and prospects on nanostructure based light emitters where active region is made with quantum-dot and quantum-dash nanostructures. Many applications ranging from silicon-based integrated technologies to quantum information systems rely on the utilization of such laser sources. Here, we link the material and fundamental properties with the device physics. For this purpose, spectral linewidth, polarization anisotropy, optical nonlinearities as well as microwave, dynamic and nonlinear properties are closely examined. The paper focuses on photonic devices grown on native substrates (InP and GaAs) as well as those heterogeneously and epitaxially grown on silicon substrate. This research pipelines the most exciting recent innovation developed around light emitters using nanostructures as gain media and highlights the importance of nanotechnologies on industry and society especially for shaping the future information and communication society.

## Abstract (web summary)

Quantum dot are one of the best practical examples of nanotechnologies. Owing to the discrete energy levels, quantum dot lasers output unique features like thermal stability, feedback insensitivity and spectral purity.

## Graphical abstract

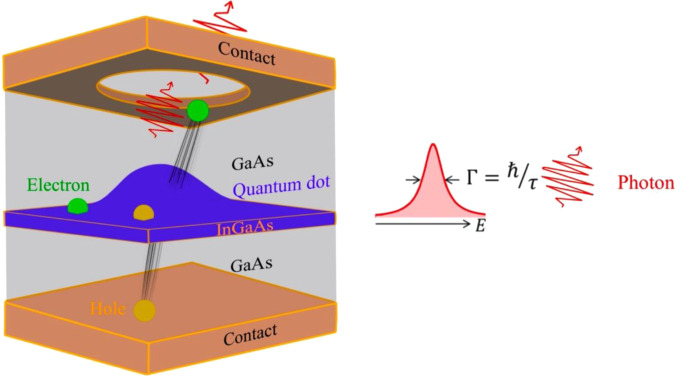

## Introduction

The economic and social development of our modern society is driven by the emergence of new technologies. For instance, the internet of things (IoT) enables the interconnection and data transmission among a plethora of physical objects such as terminal devices, vehicles, and buildings that are embedded with electronics, software, sensors, actuators, and network connectivity^1^. Without photonics, it is obvious that the interconnectedness of smart devices and subsequently their ability to share and analyze information about their surroundings is not possible^2^. In addition, the next generation of artificial intelligence (AI) based systems, specifically those using machine learning, promise strong breakthroughs going well beyond the limits of human intelligence hence creating a new class of algorithms^3^. In 5 G and 6 G optical networks, high-speed and low-latency communications enable interconnection among a wide variety of endpoints through IoT^4^. Last but not least, quantum technologies are on the way to reshape the future of internet by providing considerably faster and largely more secure data transmission owing to new encryption protocols based on quantum laws^5,6^. The rule of thumb of such key applications is that they all require the utilization of laser sources to perform complex tasks at ultra-fast speed and to enable broadband, secure and energy efficient communications^7^.

**Fig. 1:**
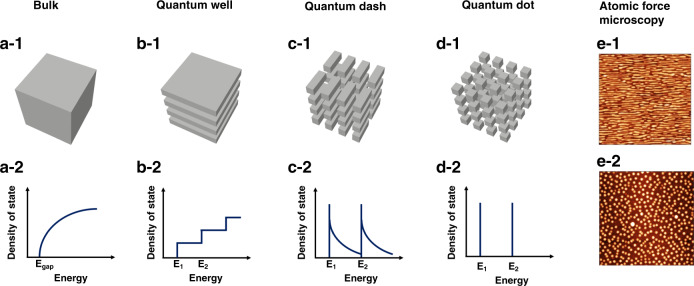
Structure and evolution of the density of states (DOS) for semiconductor structures with different degrees of dimensionality. (**a-1**, **a-2**) bulk, (**b-1**, **b-2**) quantum well (QW), (**c-1**, **c-2**) quantum dash (QDash), and (**d-1**, **d-2**) quantum dot (QD). Atomic force microscopy image of (**e-1**) QDash and (**e-2**) QD morphology

To achieve these goals, silicon photonics is one of the main driving forces offering a significant advance of the level of component integration and performance necessary for taking both classical and quantum information processing outside the laboratory^8,9^. The main motivation for pushing the development of silicon photonics is the availability of manufacturing approaches based on modern nanofabrication techniques^10^. On the top of that, integrated technologies on silicon exhibit a straightforward potential for miniaturization and integration of passive and active optoelectronic components with complementary functionalities down to the nanoscale^11,12^. In this context, quantum dots (QDs) are a class of zero-dimensional nanoclusters exhibiting an atom-like density of states (DOS). Fig. 1(a–d) reminds the structure and DOS for bulk, quantum well (QW), quantum dash (QDash), and quantum dot (QD). Thus, when one or more spatial dimensions are less than the de Broglie wavelength (~10 nm), quantization effects result in a strong modification of the DOS. In the case of QDs, the complete spatial quantization leads to an ultimate carrier confinement which enable high performance photonic devices along with possible additional quantum phenomena such as entangled-photon generation, and squeezed-state among others^13,14^. Self-assembled QD nanostructures can be formed through several growth techniques (eg., molecular beam epitaxy, metal-organic vapor phase epitaxy, metal-organic chemical vapor deposit and chemical-beam epitaxy), all of them are compatible with monolithic and heterogeneous integration on a compact and scalable platform^15,16^.

Utilizing millions of nanocrystals in the active region of semiconductor lasers was originally proposed by Arakawa et al.^17^ whereas Kirstaedter et al.^18^ and Mirin et al.^19^ showed the first clear evidence of the atom-like DOS. Since then, many research works have shown remarkable properties achieved with QD lasers leading to a new era of cost-effective optical transmitters with low power consumption and high temperature operation^15^. On one hand, InAs QDs grown on GaAs substrate are ideal for a wide range of short-reach communications (1260–1360 nm) that is to say for integrated technologies, sensing, compact laser-based radars, and high performance computing^15,20,21^. On the other hand, InAs QDs grown on InP substrates are more prone to long-reach communications (1530–1565 nm) namely for access, metro and core optical networks^22,23^. High performance InAs/InP QD lasers can be achieved, however, compared with InAs/GaAs, the formation of “true” InAs QDs on InP is more challenging since the lattice mismatch in InAs/InP (3%) is smaller than that in InAs/GaAs (7%)^21^. Such a difference along with the complex strain distribution most likely leads to the formation of elongated dots named as QDashes^24,25^. Those nanostructures exhibit a polarization anisotropy with mixed characteristics between QW and QD^26,27^. Fig. 1(e) compares the atomic force microscopy images between QDash and QD nanostructures^28^. Let us stress that both the crystallographic orientation of the nominal substrate and the growth conditions play a crucial role on the InAs/InP nanostructures morphology. For instance, round-shaped QDs can be achieved on InP(001) substrates by optimizing the arsenic species during the dot formation or by utilizing high index InP(311)B substrates^29,30^. Unfortunately, applications of InAs/InP(311)B QD lasers remain quite limited since the fabrication requires a more complex technological process which is not always compatible with the industrial standards. Finally, miscut InP(001) substrate can be considered to achieve high density of quantum wires or dots^29^.

With the rapidly progressing integration technologies, heteroepitaxy of QDs on silicon is also of vivid interest owing to the high tolerance to epitaxial defects^31^. The development of epitaxial InAs/InP QD lasers on silicon is more challenging than that of InAs/GaAs QD lasers on silicon because the lattice mismatch between InP and silicon is as large as 8% (4% between GaAs and silicon). This problem can be solved by using V-grooved silicon substrate but the defects density is still as high as 10^9^–10^10^ cm^−2^ ^32^. For InAs/GaAs QD lasers on silicon, tremendous efforts have been made to grow the QD laser on germanium-on-silicon substrate, offcut silicon substrate and on-axis (001) silicon substrate^33^. Furthermore, the epitaxial defects density has been reduced down to the order of 10^5^–10^6^ cm^−2^ and epitaxial QD lasers on silicon have recently led to record performance with low threshold current of a few milliamps, high temperature continuous-wave (CW) operation, long-lasting device lifetime along with a high yield and a much better scalability^34,35^. Besides, the use of modulation p-doping was shown to significantly improve their thermal stability and reliability^36–38^. It also reduces the linewidth enhancement factor (*α*_*H*_-factor), resulting in a high level of reflection insensitivity which is a key-point for designing isolator-free photonics integrated circuits (PIC)^39–42^. Indeed, due to the tight level of integration of multiple optoelectronic components on a photonic chip, heterogeneously integrated hybrid semiconductor lasers on silicon are more reflection sensitive^43^. Consequently, the development of feedback insensitive transmitters is of paramount importance for integrated technologies where current on-chip optical isolators do not exhibit yet sufficient isolation ratio and insertion loss^41,44^.

Digital coherent technology is also a pivotal element for optical communications including data center and access networks^45^. In principle, a coherent system is capable of retrieving both the amplitude and the phase information of optical signals, however at the price of a relative sensitivity to the phase noise of the transmitter and local oscillator^46^. In order to maintain a stable heterodyne detection, narrow linewidth semiconductor lasers are required^47^. At 40 Gbit/s, the required linewidth is to be of 240 kHz, 120 kHz, and 112 kHz for 16PSK, 16QAM, and 64QAM, respectively^48^. Apart from the coherent technology, low noise oscillators are also needed for optical atomic clocks, frequency synthesis, high-resolution spectroscopy and distributed sensing systems^49–52^. In such applications, it is important to use semiconductor lasers featuring both low frequency noise (FN) and relative intensity noise (RIN)^53^. Indeed, the RIN degrades the signal-to-noise ratio (SNR) and the bit-error rate (BER) hence affecting the performance of a high-speed communication system^54,55^. Although a low RIN floor can certainly be achieved by increasing the bias current of the laser source, it goes with an unwanted extra energy consumption. In radar applications, the RIN has also to be closed to that of the shot noise level over a bandwidth ranging up to 20 GHz hence showing the importance of generating coherent states of light for low-noise oscillators^56^. To meet these goals, QD and QDash lasers are strong candidates because they feature a low population inversion factor and reduced amplified spontaneous emission (ASE) noise^57^.

Another peculiar feature of QDs results from their large optical nonlinearities with fast response speed^58,59^. The QD gain material is spectrally broad, and the fast carrier dynamics along with the low α_H_-factor improve the four-wave mixing (FWM) based nonlinear conversion efficiency^60–62^. FWM has a great advantage in optical communications for wavelength-division multiplexing (WDM), optical clock distribution and optical wavelengths converters^63,64^. In quantum photonics, FWM can be used for having efficient quantum frequency translators and generating squeezed states of light^15^. Last, analyzing the FWM in quantum nanostructures is fundamental to understand the phase and mode-locking properties in comb lasers. Indeed, in the case of WDM systems, a mode-locked laser (MLL) featuring a frequency comb can potentially replace the large number of lasers presently necessary for the task^65^. A single-section laser using self-mode locking amplifies the advantages even further^66,67^. For instance, using InAs single section QD lasers directly grown on silicon, it is possible to achieve sufficient FWM conversion efficiency to demonstrate self-mode-locking with sub-picosecond pulse duration and kHz frequency-comb linewidth^68^.

This article reviews our recent findings in both QD and QDashes lasers and studies the connection between the material and fundamental properties that are achieved. The paper is organized as follows: Section II is devoted to discussing narrow linewidth of InAs/InP QD single frequency laser; Section III focuses on the polarization anisotropy of QDashes and investigates its impact on the reflection sensitivity of InAs/InP QDash lasers; Section IV reviews the optical nonlinear properties between QD and QDashes; Section V concentrates on the microwave photonic generation with InAs/GaAs QD lasers; Section VI analyzes epitaxial QD lasers on silicon, including material properties, linewidth enhancement factor, reflection insensitivity down to system experiments; Finally, section VII is polarized on optical frequency combs with QD lasers. The conclusion is drawn at the end of the paper. Overall, the results give a realistic frame of the device physics highlighting the pivotal role of light emitters made with QD and QDash solutions for integrated technologies on silicon as well as their possible utilization in future quantum information systems.

### Narrow linewidth

**Fig. 2:**
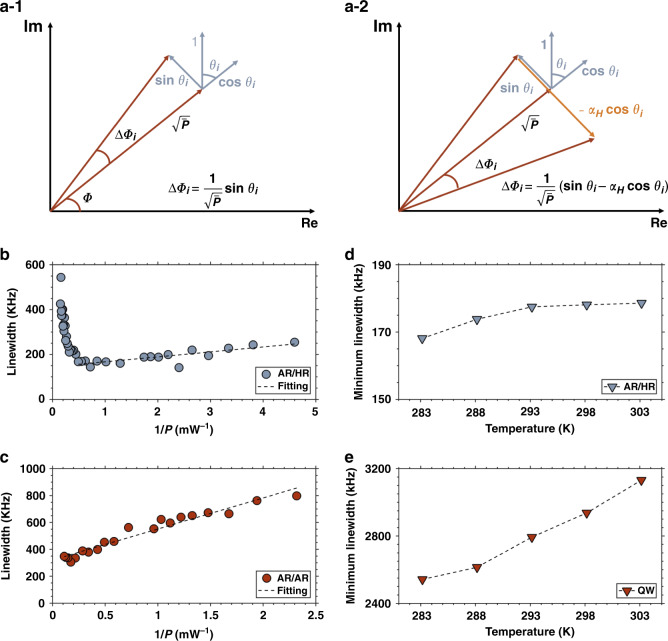
Schematic illustrating the phase diffusion ($\Delta \phi _i$) of the lasing field with (a-1) direct action of the spontaneous emission events only; (a-2) Joint action of both spontaneous emission events and phase-amplitude coupling. The angle $\theta _i$ is random. The amplitude and the phase of the lasing field are denoted ($\sqrt {\bar P}$) and ($\phi$) respectively. The corresponding phase change for each case is indicated by the relationship. Spectral linewidth fitted with a pseudo-Voigt profile versus reciprocal output power (*1/P*) for (**b**) AR/HR QD DFB laser and (**c**) AR/AR QD DFB laser. The dashed lines are linear fitting of the data. The minimum linewidth versus temperature for (**d**) AR/HR QD DFB laser and (**e**) a commercial QW DFB laser

Narrow linewidth lasers are needed in many applications such as coherent communications, spectroscopy, optical atomic clock and sensing systems^49–52^. As shown in Fig. 2(a), the spectral linewidth of laser is fundamentally driven by a phase diffusion process ($\Delta \phi _i$) which is contributed from the spontaneous emission events and the phase-amplitude coupling effect, which is described by the *α*_*H*_-factor, associated to the interaction between the intensity and phase of the lasing field. Consequently, the spectral linewidth of a semiconductor laser can be retrieved from the mean-square of the phase jitter. The corresponding equation known as the extended Schawlow-Townes relationship as follows^53^:

1 $$\Delta \nu = \frac{F}{{QP}}n_{sp}\left( {1 + \alpha _H^2} \right)$$

with *P* is the total output power, *Q∝ α*^−1^ the quality factor with *α* the total loss coefficient, and *F* a pre-factor taking into account the photon energy among others. The *α*_*H*_-factor is responsible for the much broader linewidth in semiconductor lasers whereby carrier fluctuations provide a second mechanism of phase fluctuations (see Fig. 2(a-2)) due to the phase-amplitude coupling that is to say the coupling between the carrier density, the optical gain, and the refractive index. In Eq. (1), *n*_*sp*_ is the population inversion factor that represents the minimum carrier density to achieve optical transparency. QW lasers have *n*_*sp*_ values typically between 1.5 and 2.5 while in QD lasers it is found much lower (*n*_*sp*_~1) due to smaller number of activated states needed to reach optical transparency^69^. QW distributed feedback (DFB) lasers are reliable single frequency sources, however, most of them usually have quite broad spectral linewidths of a few MHz. Eq. (1) shows that the linewidth is inversely proportional to *P* meaning that high-power lasers output narrower linewidths providing that the thermal roll-off occurring far above the threshold is mitigated. To do so, high-power DFBs are usually made with lower QW confinement and longer cavity length in comparison with high-speed DFBs. However as shown hereinafter, such structures suffer from longitudinal spatial hole burning (LSHB) hence outputting a linewidth rebroadening at high-power. To reduce the spectral linewidth below 100 kHz, different designs were proposed such as external cavity, phase-shifted and chirped grating^70^, discrete mode DFB lasers^71^, and fiber lasers^72^. The main objective is to decrease the spontaneous emission rate into the lasing mode or to increase the number of photons stored into the cavity. More recently, a III–V on silicon hybrid laser made with a harmonic potential cavity was reported. The laser unlocks record spectral linewidths owing to a high-*Q* factor resulting from the extremely low loss coefficient *α* in the silicon^73,74^.

Narrow linewidth can also be achieved by minimizing the physical quantity $n_{sp}\left( {1 + \alpha _H^2} \right)$. Here, we consider InAs/InP QD DFB lasers (cavity length *L* of 1 mm) with two types of facet coatings. The first laser used antireflection (AR) coatings on both facets with a power reflectivity below 0.01% to remove the termination phase effects of the grating at the cleaved facets. The other laser has asymmetric facet coatings, a power reflectivity of 2% on the front output facet and a high reflectivity (HR) of 62 % on the rear facet. The former laser is named as AR/AR design and the latter one is AR/HR. Fig. 2(b, c) illustrates the spectral linewidths versus the reciprocal output power (*1/P*), for the AR/HR and the AR/AR lasers. A pseudo-Voigt fitting was applied for calculating the spectral linewidth. In the low output power regime, the linewidth increases linearly with the increase of *1/P*. For the AR/HR laser, a minimum linewidth of 160 kHz is achieved at *1/P* = 0.5 transforming into an intrinsic Lorentzian linewidth of 80 kHz. In this case, the physical quantity $n_{sp}\left( {1 + \alpha _H^2} \right)$ is found as low as 1.4 at 1 mW output power against 30 for typical QW lasers^21^. Although the linewidth can be further narrowed by increasing the lasing power, it is noted that the minimum achievable linewidth is also limited by the linewidth rebroadening with the increase of output power. The spectral linewidth of the AR/HR laser rebroadens up to 544 kHz at *1/P* = 0.16. The linewidth rebroadening occurring in semiconductor lasers can be explained by thermal effects, mode instability, LSHB and gain compression^75^. The first three of these effects are directly caused by the device structure and can be minimized or eliminated through the optimization of the laser design. For instance, a low grating coupling coefficient (*κ*) is favorable for narrow linewidth operation by reducing the effects of the spatial nonlinearities. In addition, AR/AR facet coatings can eliminate the linewidth rebroadening through the control of the optical field distribution along the optical cavity. As shown in Fig. 2(c), the minimum linewidth of the AR/AR laser is at 300 kHz (110 kHz in Lorentzian shape), however, the spectral linewidth is now rather stable below *1/P* = 0.5 compared with the AR/HR laser. As the two lasers have identical dimension (cavity length, ridge width, etc.) and gain medium, the different linewidth behavior is related to the different cavity optical field distribution and the different degrees of LSHB. The field distribution of the AR/AR laser remains symmetric located at the center of the cavity and relatively weak as a result of the small normalized grating coupling coefficient (*κL* = 1). Although the AR/HR laser shows a narrower linewidth, the field distribution is much more asymmetric with enhanced LSHB effect. It is noted that every AR/HR laser may also exhibit different spectral linewidth characteristics and random facet phase effects which can be problematic for practical and industrial applications. Recently, a narrow intrinsic linewidth of 30 kHz has also been obtained in a high performance QD DFB laser^23^. Fig. 2(d, e) compares the temperature dependence of the minimum linewidth between the AR/HR laser and a commercial 1.55 μm QW DFB laser. The minimum linewidth of the QD laser is thermally stable in the range of 283 K and 303 K. The minimum linewidth of QD laser increases from 170 kHz at 283 K to 178 kHz at 303 K with 5% growth rate. In contrast, the linewidth of QW laser is increased by 23% in the same temperature range. This good thermal stability of QD lasers is a direct consequence of the tight carrier confinement and high material gain.

### Quantum dash polarization anisotropy

**Fig. 3:**
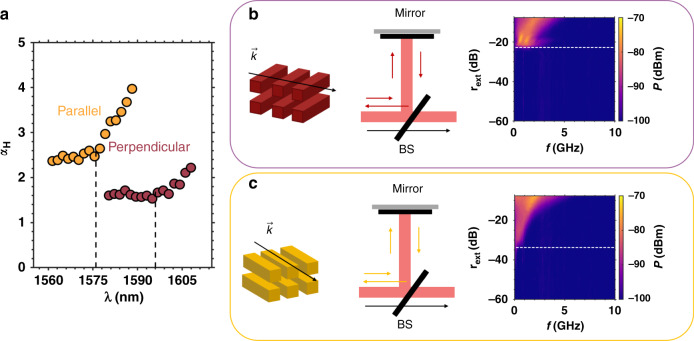
QDash polarization anisotropy. (**a**) Spectral *α*_*H*_-factor at threshold for the parallel and perpendicular QDash lasers. Sketch of the external optical feedback operation of the (**b**) perpendicular and (**c**) parallel device. $\vec k$ denotes the wave propagation direction and its interaction with the nanostructure materials that can be either perpendicular or parallel to the waveguide structure. BS beam splitter. The critical feedback level *r*_*crit*_ is marked by the white dashed line

The polarization anisotropy of QDashes was observed to largely influence the optical properties of lasers, which results from the different transition matrix element^76^. When rotating the orientation of QDashes from parallel to perpendicular with respect to the laser cavity axis, the bandgap of the nanostructures initially determined by the conduction band (C) to light hole (LH) becomes fixed by the conduction band (C) to heavy hole (HH). Fig. 3(a) depicts the dash polarization dependent spectral *α*_*H*_-factor measured at threshold at room temperature (293 K). In this study, two InAs/InP QDash devices with similar structures but having different dash orientation are investigated, the gain peak positions are marked by the black dashed lines. As shown, the gain peak of the laser with dashes perpendicular to the cavity axis (burgundy) is 20 nm longer than that of the device with dashes parallel to cavity axis (gold). This effect is attributed to the C-HH bandgap which is narrower in the former laser than the C-LH one in the latter case. Interestingly, the narrower C-HH bandgap is also beneficial for reducing the *α*_*H*_-factor of QDash lasers through increasing the modal differential gain. Here, the values of the threshold *α*_*H*_ are always found lower for the perpendicular QDash laser in the whole wavelength range. For instance, at the gain peak, the *α*_*H*_-factor is 1.5 and 2.5 for the perpendicular and parallel QDash lasers, respectively. To link the dash polarization dependent *α*_*H*_-factor to the nonlinear properties^28^, the laser’s tolerance to optical feedback is analyzed with an external fibered loop. Fig. 3(b, c) depicts the sketch of the external optical feedback operation in perpendicular and parallel devices. These figures qualitatively illustrate the light-matter interaction process between the wavevector $\vec k$ and the nanostructure materials having a different orientation with respect to the waveguiding structure. In the feedback path, light is reinjected into the laser through a back-reflector; a variable optical attenuator is used to change the feedback strength $r_{ext}$ that is defined as the ratio of the reflected power to the free-space emitting power from the front facet. In experiments, both the lasers operate in the same conditions. The lower *α*_*H*_-factor offered by the perpendicular QDash laser is beneficial for improving its tolerance for back reflections, in presence of a 11 dB increase of the critical feedback level $r_{crit}$ that is marked by the white dashed lines in the radio-frequency response. The $r_{crit}$ is defined as the $r_{ext}$ associated with the onset of coherence collapse, beyond which the laser is no longer stable against back reflections where a strong longitudinal mode broadening takes place. These results illustrate the link between the polarization anisotropy and the expected nonlinear properties. Thus, using dashes perpendicular to the cavity axis improves the laser performance in terms of reflection resistance, which is a clear guideline for designing feedback resistant laser sources.

### Optical nonlinearities

**Fig. 4:**
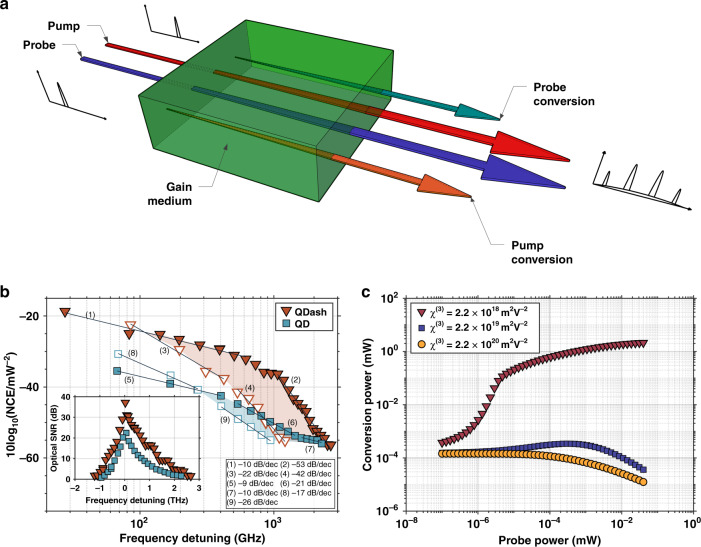
Optical nonlinearities. (**a**) Schematic representation of the four-wave mixing process in a semiconductor gain medium; (**b**) The normalized conversion efficiency NCE (solid/empty scatter–positive/negative detuning, respectively) and optical SNR (inset); (**c**) conversion power in terms of probe power for different χ^(3)^ values: 2.2 × 10^−20^, 2.2 × 10^−19^ and 2.2 × 10^−18^ m^2^/V^2^

The control of optical nonlinearities in nanostructures is meaningful not only for on-chip classical optical communications but also for quantum photonics applications whereby single-photon sources, quantum frequency converters, and squeezed states of lights are demanded^16,63^. Here, the nonlinear properties in InAs/InP QDash and QD lasers are first investigated using pump-probe experiments with dual optical injection^61^, i.e. via FWM, which is illustrated in Fig. 4(a): when two incident waves, pump and probe, enter a centrosymmetric gain medium, two additional waves are generated through third-order nonlinear susceptibility χ(3), and amplified while propagating within the gain medium. In this section, we are focusing on the conversion of the probe wave, and its normalized conversion efficiency (NCE) is shown in Fig. 4(b) along with the optical SNR presented in the inset. At low-frequency region, the nonlinear conversion is determined by the carrier density pulsation, which is directly caused by the drive-probe beating. For larger frequency detunings, carrier heating (CH) and spectral hole burning (SHB) are the dominant mechanisms taking place within sub-picosecond timescales. Overall, a NCE of -19 dB along with a frequency detuning ranging up to 3 THz is reported for the 1490 μm long QDash device in comparison to the 1250 μm long QD laser, both fabricated with metalorganic vapor phase epitaxy. Such a difference is essentially attributed to the polarization anisotropy of the QDash, particularly through the wire-like nature of the DOS (Fig. 1), which favors additional gain at the FWM-signal wavelengths hence leading to a larger conversion efficiency. In addition to that, let us stress that two-photon absorption (TPA) can further explain the larger QDash conversion efficiency, the latter was indeed found to stimulate the ultra-fast gain recovery in QDash semiconductor optical amplifiers (SOA) at energies above and below pump. Therefore, the flattening of the NCE curve observed at positively detuned pump for QDash devices around 1 THz can probably witness this phenomenon. Last but not least, it is known that carriers are captured from the bulk or QW surrounding areas into the numerous states of the dash DOS function which differs from the QD one where no energy tails occur (Fig. 1). In other words, in the case of QD, the inhomogeneous broadening is only involved between ground states hence leading to a smaller carrier coupling probability. On the other hand, owing to the optical injection, high optical SNRs of 37 dB at 27 GHz and 22 dB at 67 GHz detuning are measured for QDash and QD lasers, respectively. It is important to point out that these values are comparable with those measured for more complex DFB-laser structures^77^, bulk and QDash/QD SOA’s^78,79^, and larger than values previously reported for InAs/InP QD lasers^80^. Here, we show that larger optical nonlinearities observed with InAs QDashes grown on InP-substrate do not only result from the interaction length difference but also from TPA induced gain, faster gain saturation and enhanced modulation of carrier population, which occupy tails of wire-like DOS function inherent to QDash lasers.

In a second part, optical nonlinearities in epitaxial InAs QD lasers directly grown on silicon are investigated still with pump-probe laser experiments^68^. Using a microscopic-level multimode laser model, the relevant net FWM coefficient linked to the third-order nonlinear susceptibility χ^(3)^ is extracted^67^. It is found that the laser experiments yield clear differences in comparison with the amplifier ones, as illustrated in Fig. 4(c). Thus, for a small FWM susceptibility (χ^(3)^ = 2.2 × 10^−20^ m^2^/V^2^), mode competition overcomes the generation of the signal meaning that increasing the probe intensity depresses the signal intensity (yellow curve). With a higher FWM susceptibility (χ^(3)^ = 2.2 × 10^−19^ m^2^/V^2^), the differences arise because of the gain saturation induced by the pump and probe intracavity fields. Interestingly, there is a range of injected probe intensities where the FWM gain exceeds the attenuation from mode competition hence leading to an increase in signal intensity with injected probe power (blue curve). Eventually, the signal power drops, when the FWM gain can no longer overcome the mode competition. In case of a larger FWM susceptibility (χ^(3)^ = 2.2 × 10^−18^ m^2^/V^2^), lasing by FWM can take place, as shown by the ‘S’ shape red curve. In this case, the net FWM gain exceeds the cavity loss and is enhanced with the probe power. Here, we demonstrate that multimode laser theory provides an accurate prediction of the optical nonlinearities in the active medium and that experiments performed with lasers provide unique and valuable insights to intricate interplay of optical nonlinearities during the device operation.

### Photonic microwave generation

**Fig. 5:**
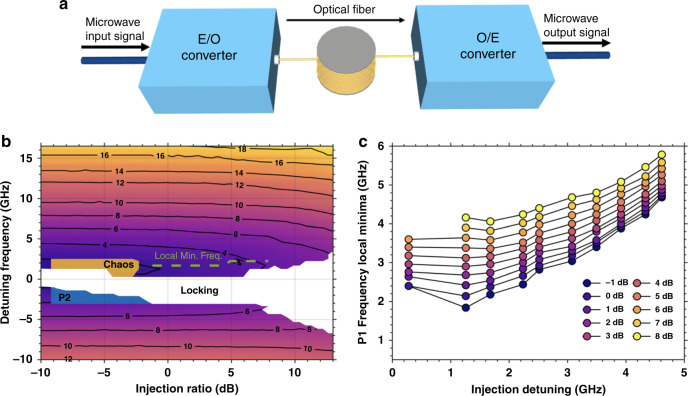
Photonic microwave generation. (**a**) Schematic of a simple microwave photonic link. (**b**) Measured microwave frequency of the optically injected QD laser. The dashes are points of local minimum frequency. The P2 and chaos regions are also displayed. (**c**) Evolution of the local minimum frequency as a function of the detuning frequency and for different values of injection ratio

Microwave signals are targeted in a wide range of applications such as antenna remoting, sensing, space communications, millimeter wave and terahertz signal generation, and cellular 5 G networks^81,82^. Fig. 5(a) shows a schematic of the key functional blocks of a microwave photonic link with. An input microwave signal is modulated onto an optical carrier through an electrical-to-optical (E/O) converter. The resulting optical signal, which carries the microwave signal, is transmitted through an optical fiber and then recovered by an optical-to-electrical (O/E) converter. Owing to the maturity of the silicon photonic platform, microwave signal generation from high performance integrated devices is also envisioned for PICs without the need of electrical modulation.

Semiconductor lasers operating under optical injection are particularly efficient for microwave generation^83^. Optical injection (OI) consists of injecting some light from a laser called master laser in the cavity of a laser called slave laser. Fig. 5(b) shows the microwave frequency map for an optically-injected InAs/GaAs QD laser with respect to the detuning frequency and the injection ratio. The former corresponds to the frequency difference between the master laser and the slave laser whereas the injection ratio is the light output from the master laser injected into the slave laser cavity. This result illustrates the potential of QD lasers for low-noise continuous photonic microwave generation displayed by the colored regime^84^. The tuneability area of the microwave signal is located from either side of the white region (stable-locking area). The microwave frequency starts with the undamping of the relaxation oscillations (~1.6 GHz for the QD laser under study), then, following the contour lines in the map, is further continuously tuned over 18 GHz by adjusting the detuning frequency. Let us stress that the chaotic and period-two (P2) oscillations displayed in Fig. 5(b) are not invoked here and their occurrence remain located on very narrow regions thanks to the large degree of stability of QD lasers^84^.

A peculiar advantage of using QD lasers is also illustrated in Fig. 5(c). The local minimum of the microwave frequency is found very stable against injection power variations. For instance, assuming an injection ratio less than 5.0 dB, the microwave frequency is nearly constant at a fixed detuning frequency, which is a relevant information for stabilizing the microwave frequency against power fluctuations originated from both the master laser and the QD laser. Here, we show that the microwave oscillation is widely tunable from the relaxation frequency up to the millimeter-wave band. High frequency microwave signals beyond 20 GHz can be achieved by considering QD lasers having very large modal gain (e.g., ~100 cm^−1^) in order to further enhance the relaxation oscillations. Also, let us stress that the large frequency detuning not only increases the microwave frequency, but also reduces the electrical second harmonic distortion and improves the optical single-side band performance (not shown here). The oscillation can also yield an intensity modulation depth of up to 100%. Last but not least, we also retrieved some particular oscillation points of low sensitivity to the frequency detuning^85^. All these insights are constructive for the development of future low-cost and energy-efficient on-chip microwave sources.

### Epitaxial QD lasers on silicon

In recent years, silicon photonics technology has been rapidly developed and commercialized, including but not limited to, datacom, telecom, optical lidars, neuromorphic computing, or quantum communications^20,39^. The primary driver in the adoption of silicon photonics is its comparably low-cost per die relative to competing technologies through its leveraging of high yield, high throughput complementary metal oxide semiconductor (CMOS) manufacturing technologies. Despite its imminent dominance, there is still ways for further improvement in manufacturability and performance by transitioning from the hybrid and heterogeneous methods of gain integration currently being deployed to a new paradigm based on direct epitaxial growth of III-V materials on silicon. To this end, it was shown that QD lasers not only provide excellent tolerance for crystalline defects, but also possess unique advantages for laser performance that cannot be matched by QWs^34^. Hereinafter, the *α*_*H*_-factor together with other peculiar dynamical features such as damping factor and relaxation frequency are discussed with the view of developing feedback insensitive transmitters.

**Fig. 6:**
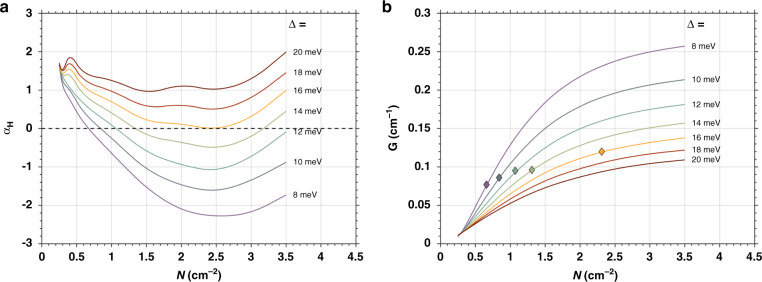
Simulated material properties of epitaxial QD lasers on silicon. (**a**) Gain peak linewidth enhancement factor (*α*_*H*_) and (**b**) peak gain as a function of carrier density (N) for ground-state transition with different inhomogeneous widths are indicated. The p-dope density is 10^12^ cm^−2^ and the diamonds show the peak gain when *α*_*H*_ = 0

Fig. 6 displays the *α*_*H*_-factor and gain calculations of epitaxial InAs/GaAs QD laser on silicon. Calculations are made with a semiclassical laser model including many-body theory. Fig. 6(a) shows that for inhomogeneous width Δ < 16 meV, a near-zero *α*_*H*_ is predicted at the gain peak (ground state transition) for certain carrier densities (*N*) whereas for Δ > 16 meV, the near-zero *α*_*H*_ is lost while minimum values still exist. The diamonds in Fig. 6(b) show the peak gains when the *α*_*H*_-factor is zero. From a parametric study, the minimum of *α*_*H*_ depends on the optimization of certain combinations of inhomogeneous width, p-doping level and carrier density. To obtain these combinations, the inhomogeneous width associated to the QD size dispersion and p-doping must be properly controlled and it can be achieved by material growth. Besides, the desired carrier density can be determined by cavity design.

**Fig. 7:**
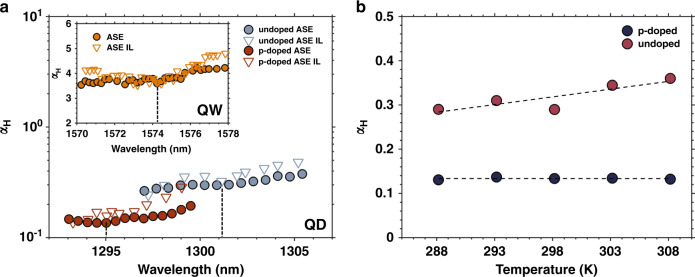
Linewidth enhancement factor (αH) measured by ASE and ASE-IL methods. (**a**) The α_H_ spectral dependence for undoped and p-doped QD lasers. The vertical dotted lines indicate gain peak α_H_ values. The inset displays the measured *α*_*H*_ values for the QW laser *α*_*H*_s. (**b**) The *α*_*H*_ at gain peak versus temperature for undoped and p-doped QD lasers. The linear curve-fittings (dotted lines) are only for guiding eyes

The experimental determination of the *α*_*H*_-factor is performed from an ASE spectroscopic analysis. This method directly measures the mode wavelength and net modal gain changes with bias current in sub-threshold operation. However, this method is not fully reliable if thermal effects are not properly eliminated since it requires a variation of the CW bias current, hence device heating usually leads to a possible underestimation of the *α*_*H*_-factor. To address this issue, the pulsed bias current was used with a minimal pulse width of 100 ns and the *α*_*H*_-factor is extracted at 0% duty cycle. Fig. 7(a) depicts the spectral dependence of the *α*_*H*_-factor for the undoped and p-doped QD lasers. For comparison, the figure in inset shows the *α*_*H*_ of a commercial QW laser. The black dotted lines indicate *α*_*H*_ values at gain peak. As shown, the *α*_*H*_-factor is of 0.30 for the undoped laser and is as low as 0.13 for the p-doped one. Those values constitute the lowest *α*_*H*_-factor value ever reported for any epitaxial lasers on silicon which are even lower than those reported on InAs/GaAs QD lasers^86^. Such impressive values result from the high quality of the material. Owing to the careful control of the threading dislocation density (TDD), it is possible to substantially reduce the inhomogeneous gain broadening and to maintain the oscillation strength near the resonant frequency. In addition, the p-doped laser has a smaller *α*_*H*_-factor than that of the undoped one which is attributed to the reduced transparency carrier density. In order to confirm the accuracy of the ASE method, the *α*_*H*_-factor is now measured by a thermally insensitive method which relies on analyzing the residual side-mode dynamics under optical injection locking (ASE-IL)^87^. As shown, the gain peak *α*_*H*_-factor of p-doped laser measured by ASE-IL is of 0.15, which is in a good agreement with that measured by ASE (0.13 at 1295 nm) with standard deviation below 15%. Not only for the p-doped laser, the *α*_*H*_-factor values of the undoped QD laser and the QW laser measured from ASE-IL are also very close to those measured from ASE method within the investigated spectral range. To sum, we found that the ASE method is well optimized and more accurate if and only if a pulse width no longer than 100 ns is used and extraction at 0% duty cycle is performed. It is worth noting that the tiny remaining difference in values between ASE and ASE-IL methods results from the fact that the ASE method still needs a variation of the bias current that causes small but persistent device heating, while the ASE-IL method requires a fixed bias current.

The effect of temperature on *α*_*H*_ is investigated both for p-doped and undoped QD lasers. Fig. 7(b) shows the comparison of gain peak *α*_*H*_-factor values as a function of temperature ranging from 288 K to 308 K. The *α*_*H*_-factor of the undoped laser slightly increases from 0.29 at 288 K to 0.36 at 308 K, which is due to the increased occupancy in the non-resonant states leading to the decrease of the ground state differential gain and the increase of the refraction index variations. On the contrary, the *α*_*H*_-factor of the p-doped laser remains stable over temperature thanks to the decrease of the Auger recombinations while those in the barrier and wetting layer increase providing rather constant refraction index variations.

**Fig. 8:**
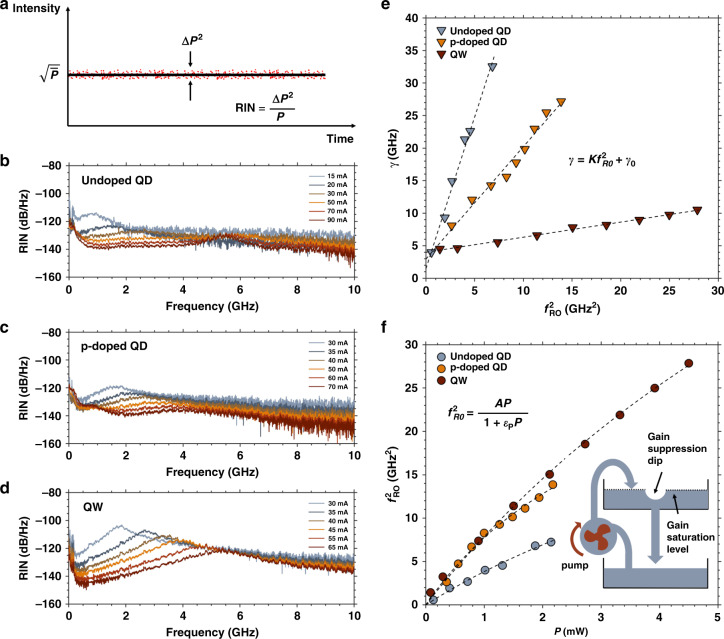
Dynamic and relative intensity noise properties. (**a**) Schematic of the relative intensity noise (RIN) of semiconductor laser. RIN spectra up to 10 GHz for (**b**) undoped QD laser, (**c**) p-doped QD laser and (**d**) QW laser. (**e**) Damping factor (γ) as a function of the squared relaxation oscillation frequency ($f_{RO}^2$) for the undoped QD laser, p-doped QD laser and QW laser, respectively. (**f**) Squared relaxation oscillation frequency ($f_{RO}^2$) versus the output power (P) for the undoped QD laser, p-doped QD laser and QW laser, respectively. The inset shows a hydraulic analogy to illustrate the gain compression in semiconductor lasers

As shown in Fig. 8(a), the RIN is defined as the ratio between the variance of the laser noise ($\Delta P^2$) and the average of laser power (P). The RIN mainly stems from intrinsic optical phase and frequency fluctuations caused by spontaneous emission and carrier noise. The dynamic and nonlinear parameters of the epitaxial QD lasers can be found from the RIN spectra through the expression^53^:

2 $$RIN\left( \omega \right) = \frac{{a + b\omega ^2}}{{(\omega ^2 - \omega _{RO}^2)^2 + \gamma ^2\omega ^2}}$$

with $\omega$ the angular frequency, $\omega _{RO}$ the angular relaxation oscillation frequency, $\gamma$ the damping factor and *a* and *b* are used for the curve fitting. Fig. 8(b–d) shows the measured RIN spectra of the undoped QD laser, the p-doped QD laser and the QW laser, respectively. The RIN spectra are recorded at various bias currents above their threshold currents while the last RIN spectrum for each laser is measured for the same coupling power (0.7 mW). The low-frequency RIN is relatively high because of the dominant contribution from current source noise, thermal noise, and mode partition^53^. It progressively decreases and saturates with the increase of the frequency and bias current. The undoped laser is overdamped with a weak peak associated to the relaxation oscillation frequency (ROF) $f_{RO}$ and minimum RIN of −140 dB/Hz at 10 GHz. By contrast, the p-doped laser has a stronger ROF peak at about 3.6 GHz, a reduced damping factor and a minimum RIN of −150 dB/Hz at 10 GHz. The QW laser exhibits even larger resonance peak at around 6 GHz and enhanced RIN of −135 dB/Hz at 10 GHz resulting from its much smaller damping factor. Fig. 8(e) shows the damping factor $\gamma$ versus squared ROF $f_{RO}^2$ among the undoped QD laser, the p-doped QD laser and the QW laser. The damping factors are growing quickly with the squared ROF for QD lasers through the relationship $\gamma = Kf_{RO}^2 + \gamma _0$ with K-factor the slope and $\gamma _0$ the inverse of the differential carrier lifetime $\tau _c$. The damping factor is found as large as 33 GHz at 3×*I*_*th*_ with a K-factor of 4.7 ns for undoped laser, while the p-doped one is reduced to 1.7 ns. On the contrary, the damping factor of QW laser rises steadily to only 10 GHz with a small K-factor of 0.2 ns. The K-factor is usually used to evaluate the maximum 3-dB bandwidth $f_{3dB,max}$ based on the expression $f_{3dB,max} = 2\sqrt 2 \pi /K$. Owing to an optimum p-doping level of 10 holes/dot, the maximum 3-dB bandwidth can be enhanced from 1.9 GHz to 5.2 GHz. As a comparison, the QW laser offers a larger bandwidth of 38.6 GHz making such transmitters much more suitable for data transmission with direct modulation of light.

As shown in the inset of Fig. 8(f), the gain compression factor describes the decrease of the gain with optical intensity in semiconductor lasers, which originates from gain nonlinearities caused by mechanisms such as CH, SHB, and LSHB^53^. The gain compression corresponds to a depletion of the gain in the upper lasing level. It is an extremely fast process (<1 ps) because of its intraband origin. On the opposite, the gain saturation is much slower (<1 ns) and related to the equilibrium between the stimulated emission and the refill of the upper level by the pump. Fig. 8(f) plots the squared ROF as a function of the output power *P* based on the following expression: $f_{RO}^2 = AP/(1 + \varepsilon _PP)$, with $\varepsilon _P$ the gain compression factor linked to the output power and *A* the modulation efficiency. From the curve-fittings, the gain compression factor for the p-doped laser is found at 0.27 mW^−1^ against 0.15 mW^−1^ for the undoped one since the p-doping introduces increased internal loss. The gain compression factor related to photon density (*S*) can be extracted from $\varepsilon _S = \varepsilon _PP/S$, where *P = hνv*_*g*_*α*_*m*_*SV* with *V* the cavity volume and *v*_*g*_*α*_*m*_ the energy loss through the mirrors. The $\varepsilon _S$ is calculated with a value of 1 × 10^−15^ cm^3^ for the p-doped QD laser against 3.1 × 10^−17^ cm^3^ for the QW one, which is consistent with previous works^21^. To conclude, using an optimum p-doping of 10 holes/dot can improve the laser’s dynamics and minimize the threshold current but at the price of higher internal loss and gain nonlinearities. All investigated dynamical parameters are summarized in Table 1.

**Table 1:** Static and dynamic parameters of the undoped QD, p-doped QD, and QW lasers

Device	undoped QD	p-doped QD	QW
Doping (cm^−3^)	0	5 × 10^17^	–
Holes per QD	0	10	–
I_th_ (mA)	13	23	28
K (ns)	4.7	1.7	0.2
$\gamma _0$ (GHz)	1.5	3.0	4.0
$\tau _c$ (ns)	0.7	0.3	0.3
*f*_*3dB*,*max*_ (GHz)	1.9	5.2	38.6
$\varepsilon _P$ (mW^−1^)	0.15	0.27	0.09
$\varepsilon _S$ (cm^3^)	5.7 × 10^−16^	1.0 × 10^−15^	3.1 × 10^−17^

**Fig. 9:**
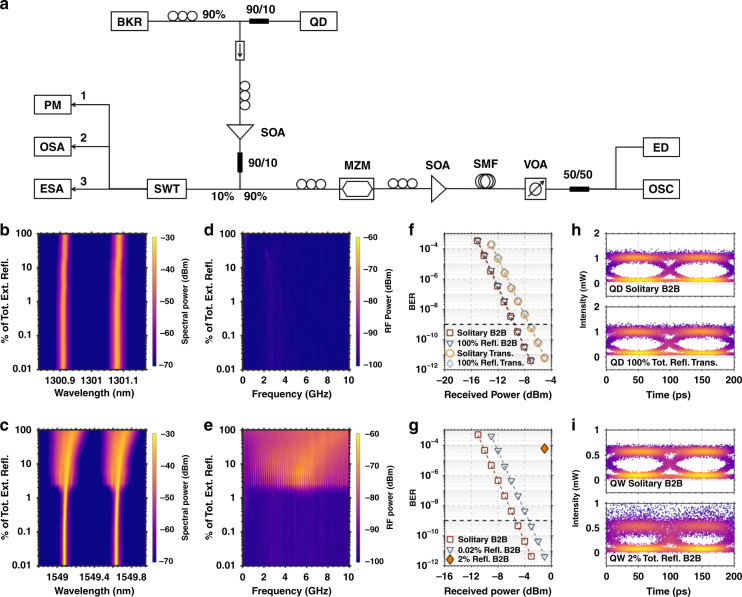
Reflection sensitivity and test-bed experiments. (**a**) Schematic of the optical feedback experimental setup. BKR backreflector, SOA semiconductor optical amplifier, SWT switch, PM power meter, OSA optical spectrum analyzer, ESA electrical spectrum analyzer, MZM Mach-Zehnder modulator, SMF single-mode fiber, VOA variable optical attenuator, ED error detector, OSC oscilloscope. Optical mappings as a function of the feedback strength at 3×*I*_*th*_ for QD laser (**b**) and QW laser (**c**). RF spectral mappings as a function of the feedback strength at 3×*I*_*th*_ for QD laser (**d**) and QW laser (**e**). (**f**) BER curves for solitary QD laser in the back-to-back configuration and QD laser with 100% of total external reflection in the back-to-back configuration, solitary QD laser after transmission and QD laser with 100% of total external reflection after transmission. (**g**) BER curves for solitary QW laser in the back-to-back configuration and QW laser with 0.02% of total external reflection. The orange triangle marker indicates the BER value with 2% of total external reflection. (**h**) eye-diagrams for solitary QD laser in the back-to-back configuration and QD laser with 100% of total external reflection after transmission. (**i**) eye-diagrams for solitary QW laser in the back-to-back configuration and QW laser with 2% of total external reflection

Owing to their large damping factor and small *α*_*H*_-factor, QD lasers are advantageous for isolator-free applications. Fig. 9(a) gives the schematic of the optical feedback experimental setup. Fig. 9(b-e) depicts the optical and RF spectral mappings of QD and QW lasers with the increase of feedback ratio *r*_*ext*_. Here, the 100% indicates the maximum feedback level in the measurements (−7.4 dB). As shown, the QD laser shows a significant tolerance against optical feedback without any mode instability, broadening and nonlinear oscillations. Conversely, the QW laser is not disturbed until the critical feedback level of 2% (−24 dB), which agrees with prior observations^88^. Above the critical feedback level, the QW laser enters the fully developed coherence collapse regime where the FP modes are strongly broadened (Fig. 9(c)) with intense chaotic oscillations occurring in the RF domain (Fig. 9(e)). In the end, high-speed experiments under external modulation at 10 Gbps are performed with and without optical feedback. Fig. 9(f) depicts that for both back-to-back (B2B) configuration and after 2 km fiber transmission, BER plots between the solitary case and for the 100% of total external reflection are perfectly overlapped, which confirms the remarkable stability against optical feedback. The eye diagrams of QD laser in Fig. 9(h) remain open both for B2B and after transmission with optical feedback, meaning that the QD laser can achieve a complete error-free operation (BER < 10^−9^) whatever the feedback conditions. On the opposite, for the QW laser, although biasing the laser in the stable regime below the critical feedback level with 0.02% of total external reflection, the BER performance is found already degraded with a 2 dB power penalty at BER = 10^−9^. The noisy eye diagram in Fig. 9(i) demonstrates that the data transmission is degraded above the critical feedback level with a corresponding BER as high as 6 × 10^−5^ (orange triangle marker in Fig. 9(g)). In summary, these results show excellent stability against optical feedback of the epitaxial QD laser, which is an important achievement for the development of isolation free transmissions on silicon chips. Such a remarkable feature results from the large damping factor as well as the high QD uniformity leading to an ultra-low *α*_*H*_*-*factor along with a robust lasing emission on the ground state transition. Last but not least, the higher TDD and epitaxial defects induced shorter carrier lifetime may also contribute to further increase the damping factor.

### Optical frequency combs

Ultra-precise comb lasers like MLLs are of particular interest for optical atomic clocks^89^, length metrology^90^, ultrahigh-bit-rate optical communication system^91^, and exhibit strong potential for new concepts in optical computing and neuromorphic photonics^92^. MLLs output a broad optical frequency comb (OFC) spectrum with a channel spacing fixed by the repetition frequency (*f*_*r*_*)* of the laser. The broad 3-dB optical bandwidth is also a strong advantage for the aforementioned applications^93^. In the time domain, the optical pulses result from the coherent addition of all spectral longitudinal modes associated to the optical cavity. Using QD lasers directly grown on silicon, mode-locking with sub-ps pulse duration and kHz OFC linewidth was recently demonstrated^66^. On the top of that, compared to a bulky DFB laser array, the compact OFC provides a solution of small foot-print and low energy-consumption. This is particularly important in WDM technologies for eliminating the energy overhead associated with independent CW lasers in the array to maintain the desired channel locking^64^. Recently, heterogeneous hybrid QD-OFCs have been demonstrated with simultaneous error-free transmission from 15 channels^94^ whereas single epitaxial QD-MLLs on silicon have shown 4.1Tb/s transmission with a 3-dB bandwidth including 58 comb lines being independently modulated^95^.

Here we investigate the optical properties of two QD-OFC lasers. The first comb device is a two-section passively QD-MLL epitaxially grown on Si. The total length is 2048 μm which corresponds to a 20 GHz fundamental repetition frequency. In addition to the gain section, a saturable absorber (SA) section is required to ensure the mode-locking operation. The second comb device is a hybrid InAs/GaAs QD-OFC laser on Si substrate. It is made of a 2.3-mm-long cavity including a 1200-μm long SOA while the laser cavity consists of a 120-μm-long SA that is placed at the center of the cavity. A 750-μm-long external cavity is positioned outside the front facet to increase the free-spectral range (FSR) to 102 GHz. The laser delivers a CW output hence without delivering pulses and the comb dynamics is activated through the reverse voltage applied to the SA.

**Fig. 10:**
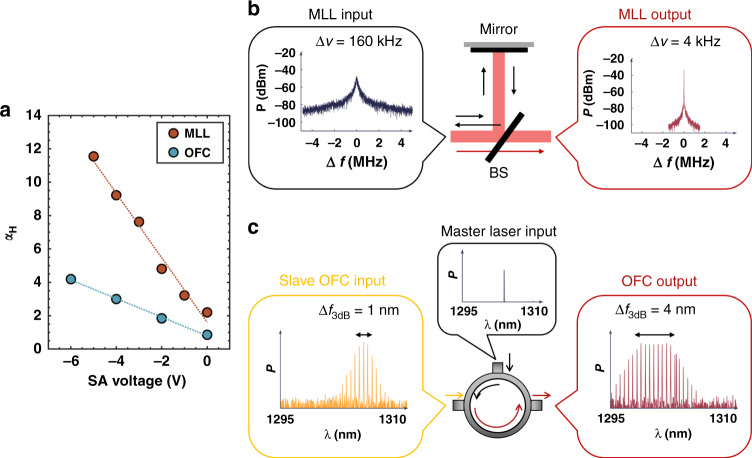
Optical frequency combs. (**a**) The *α*_*H*_ factor measured at threshold as a function of the SA reverse voltage of the MLL (burgundy) and OFC (jade). (**b**) Sketch of the optical feedback stabilization operation of a QD-MML in the frequency domain. BS, beam splitter. (**c**) Sketch of the optical injection operation of a QD-OFC

At first, we investigate the evolution of the *α*_*H*_-factor for both devices with respect to the SA reverse voltage as shown in Fig. 10(a). It is found that a larger α_H_ is always achieved with increasing the reverse voltage on the SA section both in the QD-MLL (red) and QD-OFC (blue); the larger *α*_*H*_-factor values observed from the QD-MLL are attributed to its active region made with chirped QD nanostructures. Moreover, it is worth stressing that both the SA and gain sections hold their own *α*_*H*_-factor and the contribution of each section remains to be investigated afterwards. As opposed to the uniform QDs, chirped nanostructures contribute to enhance the mode-locking performance in terms of the optical bandwidth. Then, by optically re-injecting light from an external cavity, the epitaxial QD-MLL on silicon is stabilized in presence of a large RF linewidth narrowing^96,97^. Fig. 10(b) depicts the sketch of the optical feedback stabilization (OFS) operation. The laser emission (black) firstly traverses the passive external cavity, then the returned delayed filed (gold) is coupled back to the laser cavity and it largely influences the mode-locking dynamics. In this process, a 40-fold RF linewidth reduction of the fundamental repetition frequency from 160 kHz in free-running operation down to 4 kHz is achieved, corresponding to a peak-to-peak timing jitter reduction from 60 to 9 fs/cycle. Let us stress that the RF linewidth rebroadens beyond its intrinsic value in free-running operation when the feedback loop gets out of the optimum feedback conditions. Besides, the stability of the comb operation degrades with the increase of the SA reverse bias, namely at large *α*_*H*_-factor values^98^. In this context, the QDs whose *α*_*H*_-factor is near-zero show their potential to be applied to an optical feedback stabilized MLL as the gain medium to improve its performance.

The OI technique is another controlled method that can be supplied to purify the dynamics of an OFC. Fig. 10(c) depicts the sketch of the OI operation of an OFC. The slave OFC is optically injected by an external single-mode master laser, then both the bandwidth and the flatness of the combs can be simultaneously improved by finely tuning the OI conditions, including the injection wavelength and the injection strength^65^. In the optimum conditions, the full bandwidth of the comb laser broadens from ∽6 nm (11 channels above noise floor) to ∽11 nm (20 channels above noise floor), and its 3 dB bandwidth $\Delta f_{3dB}$ enlarges from ∽1 nm (3 channels) to ∽4 nm (8 channels). Besides, a positive *α*_*H*_ is observed to enhance the OFC performance through the FWM mechanism in the active region that provides equally spaced and phase-locked modes^61^. In this case, the comb bandwidth can be enlarged though increasing the temperature-dependent homogeneous broadening of QDs^29^ or directly via the α_H_-factor through, for instance, chirped QDs^99^. To sum, the use of proper external control techniques based on optical feedback or OI can ensure a more robust comb laser operation with improved performance. The broad gain bandwidth, the tunable α_H_-factor as well as easily saturated gain and absorption make QDs good candidates for achieving precise comb diode lasers.

## Conclusion

This paper features the most recent achievements on QD and QDash lasers grown on native and silicon substrates. The connection among the material, fundamental, nonlinear, and system properties are discussed, which is crucial for achieving narrow linewidth operation, isolation-free, microwave and optical frequency comb generation. The study demonstrates the importance of using nanostructure based light emitters and highlights the impact these photonic devices have on industry and society. These laser sources can make significant advancements especially in photonic integrated technologies. Their remarkable insensitivity to parasitic reflections is unique and will enable novel functionalities and performance that were not previously achieved due to the lack of on-chip optical isolators with good performance^33,39^. The results also show that QD lasers exhibit unique properties for microwave generation while their narrow linewidth is meaningful for coherent technologies, on-chip atomic clock, and spectroscopy. Future perspective can consider deploying them in quantum technologies like for coherent and squeezing states of light^100^. In particular, squeezing states can be used to replace shot-noise-limited laser sources whereby ultralow noise oscillator operating below the standard quantum limit is highly meaningful in metrology, spectroscopy and for any precision measurements. Besides, in quantum key distribution relying on entangled photons, a large squeezing bandwidth is desirable for achieving high-speed data transmissions. Based on the results reported in this article, scientists, researchers, and engineers can come up with an informed judgment in utilizing self-assembled nanostructures for applications ranging from silicon-based integrated technologies to quantum information systems.

## References

[1] Chang, M. IoT opportunities in photonics. *Laser Focus World* **52**, ISSN 1043-8092 (2016).

[2] Yao K, Unni R, Zheng YB (2019). Intelligent nanophotonics: merging photonics and artificial intelligence at the nanoscale. Nanophotonics.

[3] Mahdavinejad MS (2018). Machine learning for internet of things data analysis: a survey. Digital Commun. Netw..

[4] Liu, X. & Deng, N. Emerging optical communication technologies for 5 G. In Optical Fiber Telecommunications VII (ed. Willner, A. E.) (Amsterdam: Academic Press, 2020), 751–783.

[5] Wang JW (2020). Integrated photonic quantum technologies. Nat. Photonics.

[6] Elshaari AW (2020). Hybrid integrated quantum photonic circuits. Nat. Photonics.

[7] Diamanti E (2016). Practical challenges in quantum key distribution. npj Quantum Inf..

[8] Asghari M, Krishnamoorthy AV (2011). Energy-efficient communication. Nat. Photonics.

[9] Vivien, L. & Pavesi, L. Handbook of silicon photonics. (Boca Raton: Taylor & Francis, 2013).

[10] Zhou ZP, Yin B, Michel J (2015). On-chip light sources for silicon photonics. Light.: Sci. Appl..

[11] Helkey R (2019). High-performance photonic integrated circuits on silicon. IEEE J. Sel. Top. Quantum Electron..

[12] Sun C (2015). Single-chip microprocessor that communicates directly using light. Nature.

[13] Song, H. Z. The development of quantum emitters based on semiconductor quantum dots. in Quantum Dot Optoelectronic Devices (eds. Yu, P. & Wang, Z. M.) (Cham: Springer, 2020), 83-106.

[14] Michler, P. Quantum Dots for Quantum Information Technologies. (Cham: Springer, 2017).

[15] Eisenstein, G. & Bimberg, D. Green Photonics and Electronics. (Cham: Springer International Publishing, 2017).

[16] Yu P, Wang ZM (2020). Quantum Dot Optoelectronic Devices.

[17] Arakawa Y, Sakaki H (1982). Multidimensional quantum well laser and temperature dependence of its threshold current. Appl. Phys. Lett..

[18] Kristaedter N (1994). Low threshold, large To injection laser emission from (InGa)As quantum dots. Electron. Lett..

[19] Mirin R, Gossard A, Bowers J (1996). Room temperature lasing from InGaAs quantum dots. Electron. Lett..

[20] Norman JC (2018). Perspect.: future quantum dot. photonic Integr. circuits APL Photonics.

[21] Crowley MT (2012). GaAs-based quantum dot lasers. Semiconductors Semimet..

[22] Liu, J. R. et al. InAs/InP quantum dot lasers and applications. Proceedings of 2018 IEEE International Semiconductor Laser Conference. Santa Fe, NM, USA: IEEE, 2018.

[23] Septon T (2019). Large linewidth reduction in semiconductor lasers based on atom-like gain material. Optica.

[24] Lelarge F (2007). Recent advances on InAs/InP quantum dash based semiconductor lasers and optical amplifiers operating at 1.55 μm. IEEE J. Sel. Top. Quantum Electron..

[25] Wan YT (2019). Low-threshold continuous-wave operation of electrically pumped 1.55 μm InAs quantum dash microring lasers. ACS Photonics.

[26] Gong M (2008). Electronic structure of self-assembled InAs/InP quantum dots: comparison with self-assembled InAs/GaAs quantum dots. Phys. Rev. B.

[27] Shi B (2020). Comparison of static and dynamic characteristics of 1550 nm quantum dash and quantum well lasers. Opt. Express.

[28] Dong B (2019). Influence of the polarization anisotropy on the linewidth enhancement factor and reflection sensitivity of 1.55-μm InP-based InAs quantum dash lasers. Appl. Phys. Lett..

[29] Even, J. et al. From basic physical properties of InAs/InP quantum dots to state-of-the-art lasers for 1.55 μm optical communications: an overview. in Semiconductor Nanocrystals and Metal Nanoparticles (Boca Raton: CRC Press, 2016), 95-125.

[30] Gilfert C (2010). Influence of the As2/As4 growth modes on the formation of quantum dot-like InAs islands grown on InAlGaAs/InP (100). Appl. Phys. Lett..

[31] Chen SM (2016). Electrically pumped continuous-wave III–V quantum dot lasers on silicon. Nat. Photonics.

[32] Zhu S (2018). 1.5 μm quantum-dot diode lasers directly grown on CMOS-standard (001) silicon. Appl. Phys. Lett..

[33] Norman JC (2021). Quantum dot lasers-History and future prospects. J. Vac. Sci. Technol. A.

[34] Norman JC (2019). A review of high-performance quantum dot lasers on silicon. IEEE J. Quantum Electron..

[35] Nishi K (2017). Development of quantum dot lasers for data-com and silicon photonics applications. IEEE J. Sel. Top. Quantum Electron..

[36] Norman JC (2019). The importance of p-doping for quantum dot laser on silicon performance. IEEE J. Quantum Electron..

[37] Duan JN (2020). Effect of p-doping on the intensity noise of epitaxial quantum dot lasers on silicon. Opt. Lett..

[38] Duan J (2018). Semiconductor quantum dot lasers epitaxially grown on silicon with low linewidth enhancement factor. Appl. Phys. Lett..

[39] Grillot F (2020). Physics and applications of quantum dot lasers for silicon photonics. Nanophotonics.

[40] Duan JN (2019). 1.3-μm reflection insensitive InAs/GaAs quantum dot lasers directly grown on silicon. IEEE Photonic Technol. Lett..

[41] Matsui Y (2021). Low-chirp isolator-free 65-GHz-bandwidth directly modulated lasers. Nat. Photonics.

[42] Huang H (2020). Epitaxial quantum dot lasers on silicon with high thermal stability and strong resistance to optical feedback. APL Photonics.

[43] Schires K (2016). Dynamics of hybrid III-V silicon semiconductor lasers for integrated photonics. IEEE J. Sel. Top. Quantum Electron..

[44] Zhang Y (2019). Monolithic integration of broadband optical isolators for polarization-diverse silicon photonics. Optica.

[45] Maniloff, E., Gareau, S. & Moyer, M. 400G and beyond: coherent evolution to high-capacity inter data center links. Proceedings of 2019 Optical Fiber Communications Conference and Exhibition. San Diego, CA, USA: IEEE, 2019.

[46] Kikuchi K (2016). Fundamentals of coherent optical fiber communications. J. Lightwave Technol..

[47] Zhang ZW (2020). High-speed coherent optical communication with isolator-free heterogeneous Si/III-V lasers. J. Lightwave Technol..

[48] Seimetz, M. Laser linewidth limitations for optical systems with high-order modulation employing feed forward digital carrier phase estimation. Proceedings of 2008 Conference on Optical Fiber Communication/National Fiber Optic Engineers Conference. San Diego, CA, USA: IEEE, 2008.

[49] Newman ZL (2019). Architecture for the photonic integration of an optical atomic clock. Optica.

[50] Spencer DT (2018). An optical-frequency synthesizer using integrated photonics. Nature.

[51] Suh MG (2016). Microresonator soliton dual-comb spectroscopy. Science.

[52] Geng JH, Spiegelberg C, Jiang SB (2005). Narrow linewidth fiber laser for 100-km optical frequency domain reflectometry. IEEE Photonics Technol. Lett..

[53] Coldren, L. A., Corzine, S. W. & Mašanović, M. L. Diode Lasers and Photonic Integrated Circuits. 2nd edn. (Hoboken, NJ: John Wiley & Sons, 2012).

[54] Duan JN (2018). Carrier-noise-enhanced relative intensity noise of quantum dot lasers. IEEE J. Quantum Electron..

[55] Zhou YG (2020). Optical noise of dual-state lasing quantum dot lasers. IEEE J. Quantum Electron..

[56] Cox CH (2006). Limits on the performance of RF-over-fiber links and their impact on device design. IEEE Trans. Microw. Theory Tech..

[57] Duan J (2018). Narrow spectral linewidth in InAs/InP quantum dot distributed feedback lasers. Appl. Phys. Lett..

[58] Akiyama T (2001). Nonlinear gain dynamics in quantum-dot optical amplifiers and its application to optical communication devices. IEEE J. Quantum Electron..

[59] Ishikawa H (1999). Applications of quantum dot to optical devices. Semiconductors Semimet..

[60] Huang H (2016). Efficiency of four-wave mixing in injection-locked InAs/GaAs quantum-dot lasers. AIP Adv..

[61] Sadeev T (2015). Highly efficient non-degenerate four-wave mixing under dual-mode injection in InP/InAs quantum-dash and quantum-dot lasers at 1.55 μm. Appl. Phys. Lett..

[62] Huang H (2015). Non-degenerate four-wave mixing in an optically injection-locked InAs/InP quantum dot Fabry–Perot laser. Appl. Phys. Lett..

[63] Stern B (2015). On-chip mode-division multiplexing switch. Optica.

[64] Cheng QX (2018). Recent advances in optical technologies for data centers: a review. Optica.

[65] Dong BZ (2019). Frequency comb dynamics of a 1.3 μm hybrid-silicon quantum dot semiconductor laser with optical injection. Opt. Lett..

[66] Liu S (2018). 490 fs pulse generation from passively mode-locked single section quantum dot laser directly grown on on-axis GaP/Si. Electron. Lett..

[67] Chow WW (2020). Multimode description of self-mode locking in a single-section quantum-dot laser. Opt. Express.

[68] Grillot, F. et al. Nonlinear-optical properties of epitaxial quantum dot lasers on silicon. Proceedings of the 28th International Symposium on Nanostructures: Physics and Technology. Virtual Event, 2020.

[69] Osborne S (2004). State filling in InAs quantum-dot laser structures. IEEE J. Quantum Electron..

[70] Zhang, Z. K. et al. 30-GHz directly modulation DFB laser with narrow linewidth. Proceedings of the Asia Communications and Photonics Conference 2015. Hong Kong, China: Optical Society of America, 2015.

[71] Kelly B (2007). Discrete mode laser diodes with very narrow linewidth emission. Electron. Lett..

[72] Pourshab N (2017). Analysis of narrow linewidth fiber laser using double subring resonators. J. Optical Soc. Am. B.

[73] Santis CT (2018). Quantum control of phase fluctuations in semiconductor lasers. Proc. Natl Acad. Sci. USA.

[74] Gallet, A. et al. Dynamic and noise properties of high-Q hybrid laser. Proceedings of 2018 IEEE International Semiconductor Laser Conference. Santa Fe, NM, USA: IEEE, 2018.

[75] Redlich C (2017). Linewidth rebroadening in quantum dot semiconductor lasers. IEEE J. Sel. Top. Quantum Electron..

[76] Ukhanov AA (2002). Orientation dependence of the optical properties in InAs quantum-dash lasers on InP. Appl. Phys. Lett..

[77] Su H, Lester LF (2005). Dynamic properties of quantum dot distributed feedback lasers: high speed, linewidth and chirp. J. Phys. D: Appl. Phys..

[78] D’Ottavi A (1997). Four-wave mixing in semiconductor optical amplifiers: a practical tool for wavelength conversion. IEEE J. Sel. Top. Quantum Electron..

[79] Lu ZG (2006). Highly efficient non-degenerate four-wave mixing process in InAs/InGaAsP quantum dots. Electron. Lett..

[80] Wang C (2014). Nondegenerate four-wave mixing in a dual-mode injection-locked InAs/InP(100) nanostructure laser. IEEE Photonics. Journal.

[81] Yao JP (2009). Microwave photonics. J. Lightwave Technol..

[82] Seeds AJ, Williams KJ (2006). Microwave photonics. J. Lightwave Technol..

[83] Qi XQ, Liu JM (2011). Photonic microwave applications of the dynamics of semiconductor lasers. IEEE J. Sel. Top. Quantum Electron..

[84] Wang C (2016). Optically injected InAs/GaAs quantum dot laser for tunable photonic microwave generation. Opt. Lett..

[85] Simpson TB (2014). Tunable oscillations in optically injected semiconductor lasers with reduced sensitivity to perturbations. J. Lightwave Technol..

[86] Huang H (2016). Multimode optical feedback dynamics of InAs/GaAs quantum-dot lasers emitting on different lasing states. AIP Adv..

[87] Wang C (2016). Thermally insensitive determination of the linewidth broadening factor in nanostructured semiconductor lasers using optical injection locking. Sci. Rep..

[88] Grillot F (2002). 2.5-Gb/s transmission characteristics of 1.3-μm DFB lasers with external optical feedback. IEEE Photonics Technol. Lett..

[89] Rosenband T (2008). Frequency ratio of Al+ and Hg+ single-ion optical clocks; metrology at the 17th decimal place. Science.

[90] Coddington I (2009). Rapid and precise absolute distance measurements at long range. Nat. Photonics.

[91] Wada O (2004). Femtosecond all-optical devices for ultrafast communication and signal processing. N. J. Phys..

[92] de Lima TF (2017). Progress in neuromorphic photonics. Nanophotonics.

[93] Fortier T (2019). 20 years of developments in optical frequency comb technology and applications. Commun. Phys..

[94] Kurczveil, G. et al. On-chip hybrid silicon quantum dot comb laser with 14 error-free channels. Proceedings of 2018 IEEE International Semiconductor Laser Conference. Santa Fe, NM, USA: IEEE, 2018.

[95] Liu ST (2019). High-channel-count 20 GHz passively mode-locked quantum dot laser directly grown on Si with 4.1 Tbit/s transmission capacity. Optica.

[96] Lin CY (2011). Microwave characterization and stabilization of timing jitter in a quantum-dot passively mode-locked laser via external optical feedback. IEEE J. Sel. Top. Quantum Electron..

[97] Verolet T (2020). Mode locked laser phase noise reduction under optical feedback for coherent DWDM communication. J. Lightwave Technol..

[98] Dong BZ (2020). 1.3-µm passively mode-locked quantum dot lasers epitaxially grown on silicon: gain properties and optical feedback stabilization. J. Phys.: Photonics.

[99] Kim KC (2010). Gain-dependent linewidth enhancement factor in the quantum dot structures. Nanotechnology.

[100] Moody G (2020). Chip-scale nonlinear photonics for quantum light generation. AVS Quantum. Science.

